# IDENTIFYING UNKNOWN STAGED CANCER IN THE UNITED STATES BY SELECTED VARIABLES DEPENDS ON THE TYPE OF REPORTING SOURCE

**DOI:** 10.1093/geroni/igae098.3998

**Published:** 2024-12-31

**Authors:** Ray Merrill

**Affiliations:** Brigham Young University, Provo, Utah, United States

## Abstract

Appropriate site-specific cancer treatment depends on the stage of disease at diagnosis. However, some cancer is unstaged, especially in older age. This study examines the level of unstaged cancer incidence for 24 cancer sites in the US according to type of reporting source and, for each source, the rate of unstaged cancer by sex, age, race, ethnicity, and household income. The rate of unstaged cancer according to cancer lethality is also investigated. Analyses were based on 2,719,800 male and 2,727,223 female malignant cancer cases diagnosed during 2015–2021. Data was collected by 22 population-based cancer registries in the National Cancer Institute’s SEER Program. Poisson regression estimated rate ratios adjusted for sex, age, race, ethnicity, and household income. Approximately 9.4% of males and 7.2% of females had unstaged cancer. The adjusted rate of unstaged cancer was 60% (95% CI 59%-61%) higher in males than females, significantly increased with age, decreased with household income, and was 11% (95% CI 10%-12%) higher in Blacks (vs. Whites) and 11 (95% CI 10%-12%) higher in Hispanics (vs. non-Hispanics). Several adjusted rate differences were identified according to the type of reporting source. Finally, there was a significant negative association between site-specific percentages of unstaged cancer and 5-year relative survival rates for males and females. Thus, the level of unstaged cancer is negatively associated with cancer lethality and depends on the type of reporting source. The type of reporting source should be considered when identifying the level of unstaged cancer according to selected variables.

